# Impact of preoperative virtual reality education on surgical patients: additional considerations

**DOI:** 10.1097/JS9.0000000000001298

**Published:** 2024-03-04

**Authors:** Zhi-Qing Zhan, Zhangkai J. Cheng

**Affiliations:** a Department of Clinical Laboratory, National Center for Respiratory Medicine, National Clinical Research Center for Respiratory Disease, State Key Laboratory of Respiratory Disease, Guangzhou Institute of Respiratory Health, The First Affiliated Hospital of Guangzhou Medical University, Guangzhou Medical University, Guangzhou; b Division of Gastroenterology and Hepatology; Shanghai Institute of Digestive Disease; NHC Key Laboratory of Digestive Diseases; State Key Laboratory for Oncogenes and Related Genes; Renji Hospital, School of Medicine, Shanghai Jiao Tong University, Shanghai, People’s Republic of China

*Dear Editor*,

We read with interest the randomized controlled study by Yang *et al*.^1^ demonstrating the potential of preoperative virtual reality (VR) education to improve disease knowledge and reduce anxiety in hepatocellular carcinoma (HCC) patients. Due to the intricate nature of liver anatomy, effectively conveying radiologic examination findings and surgical strategies to patients poses a challenge in current clinical liver surgery practices. We extend our sincere appreciation to the authors for their extensive efforts in exploring this topic. However, we believe there are key considerations that warrant further investigation to optimize the use of VR and measure its broader impact on surgical outcomes.

First, the small sample size (VR group *n*=44, control group *n*=44) and absence of blinding may compromise the reliability of the research findings. Due to the intervention’s nature, the inability to conceal the allocation of intervention from participants could have led to a placebo effect in patient-reported anxiety scores. This situation might have made the participants in the VR group more susceptible to performance bias, potentially leading to subjectively enhanced self-reports that aligned with the perceived goals of their assigned intervention during the outcome measurement^2^. Therefore, employing objective physiological measures of anxiety, such as salivary cortisol concentration or galvanic skin response, rather than relying on questionnaires would contribute to obtaining more precise results. Furthermore, the lack of blinding and the involvement of the same set of doctors in the educational intervention for both groups may give rise to bias. Specifically, the doctors’ explanations, communication styles, or areas of focus may differ between the two groups. The doctors may have also gained experience of explanation during the intervention with the first group, thereby influencing their performance when intervening with the second group, potentially compromising the independence of the intervention effects.

Second, it is important to understand that the goal of utilizing VR technology to enhance patients’ disease-specific knowledge and alleviate preoperative anxiety is to improve surgical outcomes and minimize postoperative complications, rather than solely targeting preoperative anxiety itself. While preoperative anxiety is considered a normal patient response, it may elicit a series of negative physiological and psychological reactions, thereby impacting the operation effect and postoperative recovery. Specifically, preoperative anxiety can trigger hypertension, increased heart rate, and elevated bleeding risk, affecting surgical outcomes and patient satisfaction^3^. It may also raise the risk of postoperative complications such as nausea, vomiting, respiratory distress, and cardiac events^4^. Furthermore, it is associated with heightened postoperative anxiety, increased postoperative pain, and prolonged hospital stay, ultimately impacting the quality of life for surgical patients^5^. Therefore, it is suggested that the authors include intraoperative and postoperative outcome measures such as the incidence of complications and postoperative pain scores. Investigating these measures is crucial as it not only contributes to addressing postoperative pain management issues (e.g. analgesic requirements) but is also pertinent to patients’ postoperative recovery. Liver surgery is one of the most complex procedures in abdominal surgery. The large incision during liver surgery can cause pain and sensory abnormalities for patients. Postoperatively, due to incisional pain and psychological fear of pain, patients often hesitate to get out of bed and cough, leading to an increased incidence of atelectasis and deep vein thrombosis^6^. Preoperative VR education addressing the risk of postoperative complications (such as topics 3 and 6 in this study’s VR education program) may help mitigate patients’ fear and anxiety concerning postoperative pain, enhance postoperative compliance, and reduce postoperative complications, ultimately facilitating recovery. Future research should place greater emphasis on these indicators, assessing the long-term impact of preoperative VR education on patient outcomes and the sustainability of clinical benefits.

Finally, this study restricted the population to individuals under the age of 70, and the generalizability of the intervention’s effects across different populations remains unclear. Future research should consider recruiting a larger and more diverse sample of participants across multiple sites to enhance the generalizability of study findings and the validity of the evidence generated. Additionally, the optimal timing of the intervention (e.g. before entering the operating room/during anesthetic induction) is also a worthwhile issue for exploration.

Overall, we sincerely appreciate the contributions made by Yang and his colleagues to this research, which have provided clinicians with a deeper understanding and knowledge in this field. We look forward to the authors taking these mentioned points into consideration to enhance the reliability and generalizability of their study findings, thereby advancing the better application of VR technology in the field of surgery.

## Ethical approval

Not applicable.

## Consent

Not applicable.

## Sources of funding

Not applicable.

## Author contribution

Z.-Q.Z.: original draft conception and writing; Z.J.C.: writing – review and editing. Both authors reviewed the manuscript.

## Conflicts of interest disclosure

There are no conflicts of interest.

## Research registration unique identifying number (UIN)

Not applicable.

## Guarantor

Zhangkai J. Cheng.

## Data availability statement

Not applicable.

## Provenance and peer review

Not applicable.

## References

[1] Yang J Rhu J Lim S. Impact of virtual reality education on disease-specific knowledge and anxiety for hepatocellular carcinoma patient scheduled for liver resection: a randomized controlled study. Int J Surg 2024. [Epub ahead of print]. doi:10.1097/JS9.0000000000001197 PMC11093422 38377058

[2] Wartolowska K Beard D Carr A. Blinding in trials of interventional procedures is possible and worthwhile. F1000Res 2017;6:1663. 29259763 10.12688/f1000research.12528.1 PMC5717470

[3] Hoirisch-Clapauch S. Anxiety-related bleeding and thrombosis. Semin Thromb Hemost 2018;44:656–661. 29723894 10.1055/s-0038-1639501

[4] Celik F Edipoglu IS. Evaluation of preoperative anxiety and fear of anesthesia using APAIS score. Eur J Med Res 2018;23:41. 30205837 10.1186/s40001-018-0339-4 PMC6131845

[5] Gorkem U Togrul C Sahiner Y. Preoperative anxiety may increase postcesarean delivery pain and analgesic consumption. Minerva Anestesiol 2016;82:974–980. 27028449

[6] Dieu A Huynen P Lavand’homme P. Pain management after open liver resection: Procedure-Specific Postoperative Pain Management (PROSPECT) recommendations. Reg Anesth Pain Med 2021;46:433–445. 33436442 10.1136/rapm-2020-101933 PMC8070600

